# Bio-robotics research for non-invasive myoelectric neural interfaces for upper-limb prosthetic control: a 10-year perspective review

**DOI:** 10.1093/nsr/nwad048

**Published:** 2023-02-24

**Authors:** Ning Jiang, Chen Chen, Jiayuan He, Jianjun Meng, Lizhi Pan, Shiyong Su, Xiangyang Zhu

**Affiliations:** National Clinical Research Center for Geriatrics, West China Hospital, and Med-X Center for Manufacturing, Sichuan University, Chengdu 610041, China; State Key Laboratory of Mechanical System and Vibration, and Institute of Robotics, Shanghai Jiao Tong University, Shanghai 200240, China; National Clinical Research Center for Geriatrics, West China Hospital, and Med-X Center for Manufacturing, Sichuan University, Chengdu 610041, China; State Key Laboratory of Mechanical System and Vibration, and Institute of Robotics, Shanghai Jiao Tong University, Shanghai 200240, China; Key Laboratory of Mechanism Theory and Equipment Design of Ministry of Education, School of Mechanical Engineering, Tianjin University, Tianjin 300350, China; Institute of Neuroscience, Université Catholique Louvain, Brussel B-1348, Belgium; State Key Laboratory of Mechanical System and Vibration, and Institute of Robotics, Shanghai Jiao Tong University, Shanghai 200240, China

**Keywords:** electromyography, prosthetic control, sensory feedback, EMG decomposition, robustness, deep learning

## Abstract

A decade ago, a group of researchers from academia and industry identified a dichotomy between the industrial and academic state-of-the-art in upper-limb prosthesis control, a widely used bio-robotics application. They proposed that four key technical challenges, if addressed, could bridge this gap and translate academic research into clinically and commercially viable products. These challenges are unintuitive control schemes, lack of sensory feedback, poor robustness and single sensor modality. Here, we provide a perspective review on the research effort that occurred in the last decade, aiming at addressing these challenges. In addition, we discuss three research areas essential to the recent development in upper-limb prosthetic control research but were not envisioned in the review 10 years ago: deep learning methods, surface electromyogram decomposition and open-source databases. To conclude the review, we provide an outlook into the near future of the research and development in upper-limb prosthetic control and beyond.

## Teaser

This is a perspective review of the last ten years of translational research and development efforts in non-invasive neural interfaces and robotics for practical and clinical applications of upper-limb prosthetics.

## INTRODUCTION: A DECADE OF RESEARCH

Surface electromyogram (sEMG) is the electric manifestation of skeletal muscle activations. In the literature, one channel of sEMG is usually modeled as the temporal and spatial summation of the action potentials of simultaneously activated motor units (MUs), the smallest independent functional unit of skeletal muscles. sEMG contains rich neural control information. It has been used as a non-invasive interface for bio-robotics and similar applications, including but not limited to multifunction powered limb prostheses, exoskeletons, functional electric stimulators, virtual reality and augmented reality. In a broader sense, sEMG plays a central role in establishing a non-invasive neural interface that is intuitive, convenient and accessible. Such an interface can serve as a general human–machine interface (HMI), with potential applications spanning from medical and health applications, including neurorehabilitation, diagnosis of neuromuscular disorders and physiological monitoring state of muscles (e.g. fatigue or injury), to non-medical applications, such as teleoperation of robotic devices, metaverse interfaces and active biometrics. Compared with invasive counterparts, such as intra-muscular or intra-fascicular recording methods, sEMG is not only convenient to use and poses significantly lower health risks, but also well established in practical applications outside clinical environment.

The most notable and prevalent application of sEMG is powered multifunction prostheses, often called myoelectric control. After several decades of research and development, EMG-controlled upper-limb prostheses have already become clinical and commercially viable products, serving millions of amputees worldwide. In the myoelectric control literature, there were reports of high accuracy using sEMG. For example, as early as 2005, when using only four sEMG channels, >90% decoding accuracy could be achieved in a 10-class hand/wrist gesture recognition [1]. However, one decade later, the penetration rate of commercial sEMG-controlled prostheses among amputees did not change significantly, with approximately half of upper-limb amputees abandoning the device after extensive training [2]. Obviously, there was a significant gap between research focuses in academia and the needs of the industry and end users of the devices.

In 2012, a group of academic researchers and experts from the prosthetic industry published a perspective and outlook paper examining the gap between academic research then in myoelectric control and the prosthetic industry [3]. This review paper called for a shift in research focus in this field and identified four key issues that prevented academic research from translating into industrial applications: (i) the sequential and on/off control scheme provided by pattern-recognition algorithms was not intuitive for users; (ii) lack of sensory feedback resulted in an open-loop control scheme; (iii) algorithms, in general, lack robustness against non-stationary factors ubiquitous in daily activities of living; and (iv) single sensor modality, i.e. sEMG alone, provides limited functionality and high mental load for users.

This paper, together with a more detailed subsequent review published 2 years later [4], heralded a shift in focus of the myoelectric control research community worldwide. They have become the most-cited papers in this research field in the past 10 years, with >1000 citations combined. It has been exactly one decade since the publication of the 2012 review, and the research and development landscape of myoelectric control and related topics has witnessed significant development. There has indeed been a shift of focus in academic research in all four aspects identified in the paper. It is fitting to provide a review of these activities exactly 10 years after.

One of the most significant developments in EMG processing and myoelectric control during the past years was using sEMG decomposition and decomposed motor unit action potential trains (MUAPt) as a control source. This was first proposed in the 2014 review [4] and we will also examine the development along this research direction. Further, the authors of the two review papers did not foresee the explosion of deep learning (DL) algorithms 10 years ago. The raw power of DL methods has been repeatedly demonstrated in myoelectric control literature. Studies using DL methods have shown significantly improved performance over traditional ‘feature-based’ algorithms on multiple fronts. Another aspect the authors of the two review papers did not touch on was the availability of open-source sEMG data sets. These data sets significantly reduced the barrier to conducting research in sEMG processing and myoelectric control by enabling researchers who previously did not have access to dedicated experimental infrastructure to conduct sEMG algorithm development infrastructure. Furthermore, objective and meaningful comparisons of different sEMG algorithms are made possible through testing and validation using the same data set.

Most of the past work focused on myoelectric control for trans-radial amputees, and there were fewer studies on trans-humeral amputees. One of the biggest challenges for trans-humeral amputees was the fewer residual muscles for myoelectric control, the more degrees of freedom to be controlled for the prosthesis. In this sense, sEMG alone might not be sufficient to provide enough information to restore the functionality of the neural prosthesis. Electroencephalography (EEG) signals reflected the bio-electricity of mental activities generated in the brain and are significantly less dependent on amputation conditions. Thus, brain–computer interface (BCI) or brain–machine interface (BMI) might be highly relevant to our review scope. BCI/BMI has been extensively investigated as a HMI for robotic control. A brief review of non-invasive BCI research and the hybrid of sEMG and EEG for prosthetic control in the past 10 years is also provided to complement the EMG-related body of literature.

**Figure 1.**
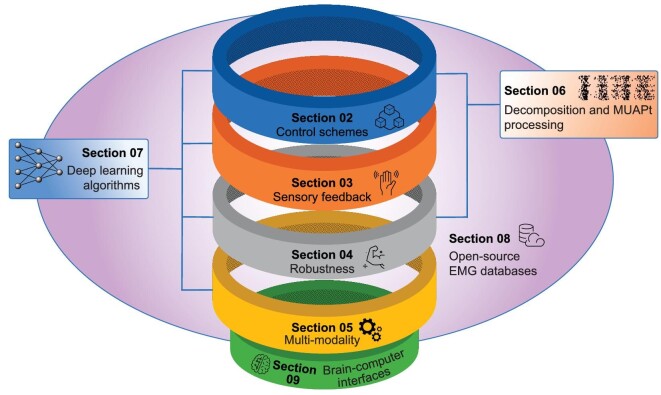
A schematic diagram summarizing the topics reviewed and their relationships.

It has been exactly one decade since the publication of the 2012 paper. It would be important to examine the progress of major research directions in myoelectric control and related areas in the last 10 years: where are we now in the effort of addressing these challenges identified 10 years ago? Are there any new challenges and opportunities emerged since then? The current manuscript aims to provide a topical summary for this objective. A schematic diagram summarizing the topics reviewed and their relationships is presented in Fig. 1. Specifically, we aim to examine whether the gap between academia and the industry, identified a decade ago, has been bridged and to what extent. Further, we tried to provide our thoughts on new research directions in these areas. The remainder of the paper is organized as follows. The research activities targeted at the four issues identified in the 2012 paper are examined, followed by a review of EMG decomposition and myoelectric control research based on MUAPt analysis. Next, we provide a dedicated section on the literature on using DL in various aspects of myoelectric control research, as well as a section summarizing recently emerged open-source sEMG databases and their impact on the research literature. Before the conclusion section, we present a brief review of relevant non-invasive BCI research. We finally conclude the review by providing an outlook into the near future. This review was not meant to be an exhaustive review of all research activities on this topic that has emerged over the past decade and there might be unintended omissions of important and relevant work, for which the authors express their sincere apologies.

## CONTROL SCHEME: TOWARD INTUITIVE AND NATURAL CONTROL

The first issue identified in the 2012 review and further elaborated on in the 2014 review is the overall myoelectric control scheme. There are mainly three different control schemes: traditional direct control (tDC), pattern-recognition-based control (PRC) and simultaneous and proportional control (SPC). Table 1 illustrates the pros and cons of EMG-based prosthesis control approaches.

**Table 1.** Pros and cons of EMG-based prosthesis control schemes.

Control scheme	Traditional direct control (tDC)	Pattern-recognition-based control (PRC)	Simultaneous and proportional control (SPC)
Proportional or discrete control	Proportional	Discrete	Proportional
Sequential or simultaneous control	Sequential control, single DoF at a time	Sequential control, actuating one motion at a time	Simultaneous control of multiple DoFs
Switching commands	Non-intuitive switching commands	None	None
Intuitive control	Non-intuitive, require mental and physical efforts	Non-intuitive, unnatural way of performing tasks	Intuitive, enable more natural control

### tDC

To date, a vast majority of the commercially available prostheses are controlled via tDC. In this control approach, the speed of a motor in one direction is proportional to the magnitudes of EMG signals recorded from one agonist–antagonist muscle pair [5], requiring two independent EMG channels to control one degree of freedom (DoF) (e.g. hand open/close). As such, the user must generate a special contraction (e.g. a co-contraction) to control a different DoF (e.g. wrist flexion/extension). The tDC requires significant physical and mental effort from the user, resulting in a limited clinical and commercial impact of myoelectric prostheses: only one-fourth of upper-limb amputees use prostheses with daily life [6].

### PRC

To address the above limitations of tDC, myoelectric control based on PRC has been widely investigated for decades, particularly on trans-radial (TR) amputees. In this scheme, the controller uses a set of EMG algorithms to identify the user's intended motion or gesture using EMG acquired from the residual limb muscles, while amputees perform the motion of the phantom limb. According to the decoded motion or gesture, the controller actuates the corresponding motor(s) and controls the speed of the motor in proportion to the overall magnitudes of EMG signals. Compared to the tDC, PRC allows the user to articulate a set of predefined motions or gestures of the hand/wrist. As discussed in the 2012 and 2014 reviews, PRC provides better functionality and controllability than tDC.

PRC has been an active research direction for the past 10 years and commercial products embedded with PRC became available from Coapt Pattern Recognition [7] and Myo Plus Pattern Recognition [8]. The focus of this line of research, though, has shifted significantly. Before the 2010s, most myoelectric control studies on PRC pursued classification accuracy using a given data set, usually obtained in well-controlled laboratory conditions. In the last decade, increasingly more studies have aimed at improving the robustness of the controller in the presence of various non-stationary factors and external disturbances.

In 2013, Li *et al*. developed an EMG PRC scheme based on boosting and random forest classifiers [9]. The probability of the prediction was adopted to balance the correct classification and the rejection of the untrained motions. The experimental results demonstrated that the proposed PRC scheme was robust to untrained motions and had great potential for practical applications. In 2016, Liu *et al.* proposed a domain adaptation (DA) framework reusing the models trained by data from earlier sessions as baseline models to be adapted with newly available data [10]. The DA framework could potentially reduce the calibration time and significantly improve session-to-session robustness. In this sense, this is probably one of the earliest transfer learning studies in myoelectric control literature. In 2017, Khushaba *et al.* employed time-domain descriptors to estimate the power spectrum characteristics of the EMG signals [11]. The proposed temporal-spatial descriptors were evaluated on multiple conventional and HD-sEMG data sets. The classification performance of the methods was significantly superior to other classic methods. In 2019, Behrenbeck *et al.* proposed using spiking neural networks (NeuCube model) to extract the spatio-temporal information from raw sEMG signals instead of using hand-crafted features [12]. The results demonstrated that the NeuCube model outperformed conventional PRC approaches. In 2020, Xiao *et al*. converted the original sEMG signals into Gramian Angular Summation/Difference Field (GASF/GADF) images and then extracted the Histogram of Oriented Gradient (HOG) features of the corresponding GADF and GASF image [13]. The six motion classification accuracies of the GASF-HOG and GASF-HOG were 93.63% and 95.73%, respectively. Lv *et al.* recently proposed a signal processing method for identifying hand motions from sEMG using a self-organizing mapping network for feature extraction and a radial basis neural network for pattern classification [14]. The average classification accuracy of eight gestures across six subjects attained 96.875%. In 2022, Xiong *et al*. proposed a new feature extraction method to extract spatial structural information from sEMG signals through the symmetric positive definite (SPD) manifold [15]. The proposed method characterized non-Euclidean representations embedded in sEMG signals for PRC applications. The proposed SPD manifold-based method with an support vector machine (SVM) classifier achieved the classification accuracy at 84.85%.

Many DL algorithms have been investigated for PRC. They are discussed in a dedicated DL section, along with DL studies for other aspects.

### SPC

The two reviews in 2012 and 2014 advocated for SPC as a necessary feature of intuitive myoelectric control. Indeed, SPC has drawn considerable attention from the research community in the effort to provide more intuitive and natural control for prosthesis users in the last 10 years. The decoding algorithms for SPC can be categorized into three research directions: unsupervised, supervised and musculoskeletal model-based methods. Similarly to the PRC, DL-based SPC studies are not presented here but are discussed in the dedicated section.

#### Unsupervised SPC methods

Following the original non-negative matrix factorization (NMF) SPC study in 2009 [16], Jiang *et al.* further demonstrated the feasibility of the proposed NMF-based method for online SPC of two wrist joint DoFs on TR amputees [17]. In order to further reduce the user's calibration effort, Lin *et al*. proposed a sparseness-constrained NMF (SNMF) [18]. SNMF uses data from arbitrary multi-DoF contractions as training data rather than the restrictive single-DoF contractions in the previous method. The accuracies of NMF-based algorithms are still limited, partly due to their unsupervised nature. In real-world applications, particularly for unilateral amputees, joint angles and/or other biomechanical information from the intact side can be used as the training label. As such, supervised regression methods have also been investigated extensively.

#### Supervised SPC methods

Muceli *et al.* proposed a multi-layer perceptron (MLP) network for SPC of multi-DoFs of the hand and wrist [19]. This study demonstrated that complicated hand and wrist movements could be predicted from the high-density (HD) EMG signals of the contralateral limb. The proposed approach is feasible for practical applications of supervised SPC on unilateral TR amputees. Hahne *et al*. demonstrated the feasibility of predicting two-DoF wrist movements from EMG signals by regression techniques [20]. The study compared four linear regression (LR) and one non-linear regression technique for EMG-based SPC of two-DoF wrist movements. Ameri *et al*. realized the multi-DoF online SPC based on SVMs [21]. The study compared the online performance of the SVM-based approach with that of the MLP-based approach. The online performance was quantified by completion rate, path efficiency and overshoot times. The results demonstrated that the SVM-based approach achieved better performance on all metrics and significantly reduced the processing time compared with the MLP-based approach.

#### Musculoskeletal model methods

In recent years, the feasibility of realizing SPC based on EMG-driven musculoskeletal models (MMs) has been investigated by several research groups [22–27]. Instead of exploring data structures, the EMG-driven MM incorporates the anatomical and biomechanical structures/constraints of the neuromusculoskeletal system of the human limb. By explicitly representing the anatomical and biomechanical structures/constraints, the MM can better mimic the physiological generation process of human movements. Therefore, MM methods better decipher continuous and coordinated movements from sEMG compared with the aforementioned data-driven approaches. Furthermore, the MM has shown inherent reliability across different upper-limb postures [28] and loading weights [29].

In 2016, Crouch *et al*. developed an EMG-driven MM for SPC of two-DoF hand and wrist movements [22]. The structure of the model was simplified from a comprehensive model to be clinically feasible. In 2017, Blana *et al*. proposed a 23-DoF MM of the hand [23]. This model used extrinsic muscles and realized the mapping of EMG and hand movements in real-time simulation. In 2018, Pan *et al*. developed a generic EMG-driven MM for online SPC of two-DoF hand and wrist movements [24]. The study demonstrated that the generic MM could sufficiently model newly recruited able-bodied and TR amputee subjects, and was reliable across different upper-limb postures. The generic MM has great potential to solve the challenge of frequent and lengthy calibrations of existing EMG interfaces. In 2018, Sartori *et al*. proposed a three-DoF EMG-driven MM for online SPC [25]. The proposed three-DoF MM consisted of 10 hand and wrist muscles. The results showed the effectiveness of the three-DoF MM on both able-bodied subjects and a TR amputee. In 2020, Blana *et al*. investigated the capability of the MM for finger movement control [26]. The kinematic fidelity of the estimated movements and the computational performance of the MM were evaluated. The results indicated that the MM is promising for real-time prosthesis control.

### Outlook

Although the EMG PRC and SPC-related studies have gained tremendous momentum in the past decade, the EMG-based interface still has great potential for improvements in the following aspects. Since two PRC-based myoelectric prostheses were commercialized in the last decade, namely Coapt [7] and Myo Plus [8], the research in PRC-based EMG interfaces should accelerate its shift towards practical and clinical applications, such as improving the robustness against the non-structural environment, reducing the user's training effort and providing better user embodiment of the prosthesis. To achieve natural and intuitive control, SPC-based myoelectric control would still be the research hot spot in the future. The number of DoFs investigated in SPC-based EMG interfaces was still limited to the wrist. Exploring the potential of developing the SPC with higher DoFs, potentially those of fingers, to mimic dexterous human hand movements would attract more researchers’ attention. Given that the MM-based approaches demonstrated promising results in myoelectric control, incorporating the anatomical and biomechanical structures of the neuromusculoskeletal system while developing EMG-based interfaces in the future would not only improve the performance and robustness of the interface but also be helpful in understanding the physiological processes from the neural drive (EMG) to the executed movements. Recently, Laffranchi *et al*. showed with advanced anthropomorphic design that it is possible to realize SPC by a novel direct control approach [30].

## CLOSING THE CONTROL LOOP: SENSORY FEEDBACK

In a narrow sense, providing sensory feedback is not part of myoelectric control. However, when examining overall prosthetic control, forward motor control (driven by EMG) and sensory feedback are two closely coupled parts of the sensorimotor loop—the Yin and Yang of the Taichi. However, the state-of-the-art in prosthetic sensory feedback is significantly lagging behind its forward control counterpart. The amount of research is comparatively limited in both range and depth. Not surprisingly, no commercially available prosthesis provides somatosensory feedback functionalities, either tactile or proprioceptive. Without the sensory feedback, the ability for fine control of a prosthesis is limited and the amputee user must rely heavily on visual feedback to perform the functional tasks, inevitably inducing a high cognitive and psychological burden. Furthermore, the absence of sensory feedback impedes the functionality and efficiency of dexterous hand prostheses, which is likely to play an important role in prosthesis rejection rates.

As identified in the 2012 review, the lack of sensory feedback of upper-limb prostheses is a key challenge in prosthetics research. The key challenge is that the sensory pathway cannot be directly established between the prosthetic hardware and the human sensory system. Thus, the sensing information retrieved from sensors embedded in the prosthesis can only be transferred to amputees by eliciting the remapped physiological sensations through relaying stimulation mechanisms. Specifically, it requires sensing a variety of tactile (e.g. pressure and vibration) and proprioceptive (position and velocity) information, as well as communicating to amputees the information through non-invasive or invasive neural interfaces.

### Feedback techniques

The sensory nerve endings of the human hand are connected with four classes of mechanoreceptors in the skin (Merkel disks, Ruffini cylinders, Meissner corpuscles and Pacinian corpuscles), responding to various tactile sensations and skin deformation [31]. Nerve fibers that innervate the various mechanoreceptors in the glabrous skin of the hand converge to form three nerves, namely the median nerve, ulnar nerve and radial nerve, which reciprocally innervate five digits. The artificial sensations are evoked by stimulating the spared tactile sensory structures using non-invasive (mechanical or electrotactile) and invasive (direct nerve and sensory cortex stimulation) interfaces.

**Figure 2.**
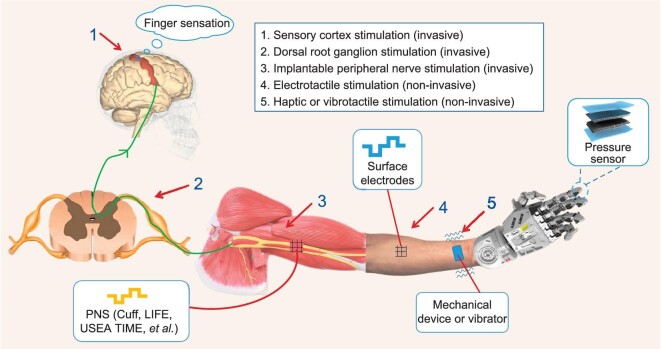
Schematic diagram of sensory feedback for prosthetic control.

Upper-limb amputation results in the loss of the mechanoreceptive end organs. However, the somatosensory nerves and ascending pathways conveying signals from those organs to the central nervous system remain functional. Nevertheless, the natural hand–object interaction generally activates hundreds or even thousands of tactile nerve fibers and the activation patterns are task-dependent. It is impossible to replicate with high fidelity such complex ‘encoding’ processes in the foreseeable future. As such, the sensory restoration of neural prostheses is limited by a minute number of stimulating channels relative to the naturalistic number of nerve fibers. Therefore, the goal is not to reproduce the natural sensory feedback with high fidelity. Given the limited stimulation channels, the goal is to produce patterns of neuronal activation that are useful for object manipulation and promote the embodiment of the prosthetic hand. According to different neural interface techniques, somatosensory feedback can be divided into mechanotactile, electrotactile and invasive sensory feedback, as shown in Fig. 2. Here, we only focus on the non-invasive interface for sensory feedback. Please refer to [32] for an excellent review of invasive methods.

### Mechanotactile feedback

Mechanotactile stimulation can deliver modality-matched feedback from a prosthetic hand to its user. It has been shown that multiple stimulation sites and pressure intensities could be effectively transferred by mechanotactile devices. However, mechanotactile feedback is hard to integrate into the socket of a prosthetic hand due to its bulky volume and heavy structure [33]. As a form of mechanotactile feedback, vibrotactile feedback is generated by applying a set of vibrators on the surface of the target skin area. Some commercial myoelectric prostheses, including i-Limb, Cyber-hand, MANUS-hand, Fluid-hand and Smart-hand, have been used to validate the improved efficiency in prosthetic control by incorporating vibrotactile feedback [34].

Tactile information can be encoded by changing frequency, amplitude and location [35]. Close-loop force feedback was implemented by encoding the contact force between the prosthesis finger and objects via vibration [36]. Some studies adopted vibrotactile stimulation to encode texture information of grasped objects by varying vibration frequencies [37]. It was shown that the proprioception sensation could also be successfully elicited when vibrators are located near the tendon in a range of frequencies (40–80 Hz) [38].

To increase the bandwidth of the vibrotactile feedback pathway, researchers have developed multichannel vibrotactile devices with arrays of vibrators [39]. The stimulation stability of vibrotactile feedback is influenced by the variation in contact stress and ever-changing perception threshold. The performance of vibrotactile feedback is affected by factors such as stimulation locations, different thresholds and varying levels of cognitive ability across subjects. In addition, unpleasant noises will be generated during vibration. Moreover, single modality vibrotactile feedback cannot meet the modality matching requirements in continuous contact situations [33]. In conclusion, vibrotactile feedback is a method that can enhance the grasping performance of prostheses despite the many limiting factors listed above.

### Electrotactile feedback

Transcutaneous electrical nerve stimulation (TENS) (also called electrotactile stimulation) has been considered a viable non-invasive approach to restore sensory feedback, encoding tactile information from the prosthetic device and providing them to amputees. It is extensively adopted due to its advantages of multiple adjustable stimulation parameters (e.g. pulse amplitude, pulse width and frequency), fast response and low noise. Evidence also suggested that TENS not only can reduce phantom pain and stump pain caused by amputation [40], but can also induce a sense of perceptual embodiment in able-bodied subjects with a visual–tactile illusion and in amputees with stimulation of sensory afferents [41]. It has been shown that multiple types of tactile sensations (such as pressure and vibration), referred to as finger-specific sensations, can be elicited through electrotactile stimulation [42]. The features of a grasped object (e.g. shape, size and stiffness) can thus be encoded and delivered via electrotactile feedback using multiple encoding schemes [43]. Close-to-natural finger sensations were transferred by stimulating the phantom finger map that formed as a result of amputation and it was proved that amputees with a complete phantom finger map could identify the stimulated patterns and areas with >90% accuracy [44]. A non-invasive, bidirectional prosthesis based on TENS was implemented, in which electrodes placed on the skin were able to activate underlying nerves and generate conscious sensations referred to the phantom limb [45]. Subjects were asked to perform several functional tasks to validate the potential increase in prosthesis use performance offered by the added sensory information.

Electrotactile feedback is a potential method for the sensory restoration of advanced prostheses. However, implementation of the closed-loop prosthesis still faces many unsolved issues, e.g. miniaturization of the stimulation device and skin-impedance fluctuations.

### Proprioceptive feedback

Compared with the functional reconstruction of tactile feedback, there is a bigger challenge to realize the matching between proprioceptive motion information of prostheses (including joint angles of the missing limb) and the proprioception perception of amputees. One group of techniques consists of sensory substitution methods incorporating other modalities (tactile, vibration, skin stretch) for implementation of the position and the force (exerted by the prosthesis) feedback, which is closely related to the topic of non-invasive sensory feedback [46]. However, these techniques can hardly be called intuitive as they involve the transformation and rerouting of sensed information from the prosthesis to an available sensory channel of the user (potentially of a different modality) at a free site of the stump and require extensive training. Additionally, these techniques cannot engage remnant muscle proprioceptors, which are important for natural kinesthetic perception. Other developing techniques enabling connection to the neural periphery have confirmed that near-natural proprioceptive sensations can be provided through invasive neural technologies at various levels in the peripheral or central nervous system, which can be referenced in a recently published review [47].

### Outlook

Currently, advanced upper-limb prostheses can provide meaningful compensation for motor function loss. However, their ability to compensate for sensory function loss is comparatively limited. Technical challenges exist in many aspects, such as the integration of sensors, the portability of stimulation devices and the establishment of a neural sensory pathway between the prosthetic hand and the human sensory system. For these purposes, multimodal sensing capability (e.g. pressure, vibration and temperature) and high-density sensor units are desirable to provide better interaction experiences with different objects. In addition, more portable and compact stimulation feedback hardware with advanced encoding strategies must be developed to transfer the sensing information retrieved from the prosthesis to the amputee in an untethered prosthetic hand system.

## GETTING OUT OF THE COMFORT ZONE: ROBUST MYOELECTRIC CONTROL

One of the prevailing assumptions in myoelectric control algorithm development was that the features extracted from EMG are sufficiently stationary for the same motion or gesture. While it certainly holds in controlled laboratory conditions, this assumption is no longer valid with data from real-world scenarios, where many factors introduce non-stationary disturbances into the data. These factors can be categorized into two groups: internal and external. The internal factors are from the user, including muscle fatigue, limb/trunk position change, muscle contraction intensity change and others. The external factors are from sources other than the user, including a shift in the electrode positions, electrode–skin-impedance changes, electrode faults and more. Since the 2012 review, improving robustness against these factors has been one of the key topics in myoelectric control research. Of these factors, electrode shift, muscle fatigue, muscle contraction intensity change and upper-limb movement received the most attention.

### Solutions

Retraining the model using the most current data is the most straightforward way to restore performance against non-stationary factors. However, frequent retraining is time-consuming, making the interface inconvenient and impractical. Alternatives were proposed for different factors and they could be classified into four types, i.e. data augmentation, invariant feature extraction, user training and sensor fusion.

Data augmentation refers to obtaining additional training data for designing algorithms to improve generalization of the model. Two key differences from retraining should be noted: (i) the additional data for augmentation are obtained under different conditions from the original training data, e.g. such as electrode position; and (ii) the original model was adapted rather than not completely abandoned. Data augmentation methods could be categorized based on how data fusion is performed. A common way is to fuse information at the feature level, i.e. group training. For example, for the factor of electrode shift, in addition to the original positions, the electrodes would be positioned to the potential shifted locations successively for data collection. The features of the same gesture were labeled as the same class and combined as training data. The method was effective in reducing the influence of most disturbances. However, with the increase in the training data, the feature space would be crowded and the improvement would be diminished [48]. Even worse, the performance in the original condition could also be affected [49]. Other than the feature-level fusion, there are algorithms to update the parameters of the original model using the additional training data, which is normally called domain adaptation or transfer learning. Generally, the algorithms aim to tune the original model with the insufficiently labeled data in an efficient way. As such, differently from group training, the amount of additional data is significantly lower than that of the original data. It has been applied to the issue of electrode shift [50] and stability of the between-day performance [51], and the performance significantly improved after the disturbances [52]. The algorithms could also be combined with deep learning frameworks for better robustness, which was presented along with other deep learning contents (see `DL for decomposition and MUAPt mapping' Section). Recently, instead of tuning the model parameters, He *et al.* proposed an alternative approach to fully restore the performance by interactively guiding the user to turn the electrode position back to its original position with the additional data from just one anchor gesture [49], which provides a different angle to utilize the information of the additional data.

Invariant feature refers to signal features that are invariant to the disturbances. Unlike data augmentation, which can be considered ‘more knowledgeable’, the invariant feature approach can be considered ‘smarter’ [53]. The advantage is clear: there is no need for additional data. The required features should be invariant to the non-stationary factor to achieve this goal. Because different non-stationary factors affected different aspects of the signals, the invariant features to be extracted would be different. For example, because force variation mainly affects the signal amplitude, the force-invariant features should not be extracted from the amplitude [54]. For electrode shift, spatial filtering was employed to extract information invariant against electrode position change in both the PRC approach [55,56] and the SPC approach [57]. The invariant feature is the ideal solution for robust myoelectric control, but it should be noted that the current proposed invariant features are not truly invariant against the disturbances but can reduce their influences. Though the performance in the presence of disturbances could be improved to some extent, it could not be fully restored. Further, it would be difficult to find an invariant feature against multiple disturbances, which usually co-exist in practical scenarios.

User training refers to training the user to improve the signal quality following performance degradation due to non-stationary changes in the signal. Differently from the first two approaches, this strategy tackled the problem from the user's perspective instead of the algorithm. It was shown that no statistical difference was found between with-day and between-day performance after 1 week of open-loop training, highlighting the inherent ability of the users’ adaptation [58]. With that, effective training methods with feedback were proposed to further accelerate this process [59] in the PRC approach. These studies demonstrated that user training and adaptation were important in enhancing the robustness of the interface, especially in maintaining the performance in long-term use (e.g. across multiple days). In addition, in the SPC control scheme, users could adjust their contractions based on the visual feedback to adapt to the model change from the disturbances [60]. This evidence demonstrated the importance of the user factor in myoelectric control.

Sensor fusion refers to introducing signal modalities other than sEMG to provide complementary information. The effects of the disturbance on different signal modalities were different and they could be combined to improve the control performance with the disturbance. This part is presented in the next section, along with other multimodality studies.

### Outlook

Though the strategies mentioned above were, to various degrees, effective in improving overall system robustness, the reported system performance in the presence of disturbances was still lower than the original state, indicating the control would still be affected. Further, many studies focused on the disturbance of only one factor. However, in the real world, multiple factors usually occur simultaneously. It was necessary to investigate multi-factor scenarios and develop robust solutions for them [61].

The current performance evaluations are mostly conducted with offline analysis or computer-based online virtual assessment and the disturbances were mostly introduced artificially. Since the final goal of the interface was the practical applications, evaluations with the real-world function should be performed, such as the box-and-blocks test and the clothespin test while wearing the real functioned prosthesis.

## MULTIMODALITY SENSING: 1 + 1 > 2

### More than just EMG

Although myoelectric signals contain rich neural control information of human movements, they may not be sufficient to decode the intentions of human movements alone accurately. As such, the 2012 review calls for sensor fusion methods to further improve the control performance and functionality of the prosthesis. Similar to the first three challenges, this challenge has received considerable attention over the last decade. Various methods, such as mechanomyography (MMG), force myography (FMG), inertial measurement unit (IMU), infrared spectroscopy (NIRS), ultrasound (US), EEG and computer vision (CV), have been investigated. Some modalities were fused with sEMG to improve the robustness against the disturbances. For example, MMG and FMG would not suffer from the skin-impedance change, and MMG was also insensitive to the sensor position [62]. IMU could be used to predict the position of the upper limb, which is beneficial for eliminating its adverse effects [63]. NIRS could measure the state of muscles to predict fatigue [64]. The other modalities were fused to improve the control performance. For example, US has high spatial resolution and can detect deep muscle activities. It was shown that there were complementary advantages of A-mode US and sEMG in gesture recognition [65]. As we stated in the introduction, EEG was less dependent on the amputation conditions, which rendered it an excellent candidate for providing a tangible prosthetic control solution for elbow-above amputees. We will dedicate a separate section to discuss BCI and its potential to be fused with sEMG. For the fusion algorithms, the data could be fused at the feature level, in which the extracted features were integrated to train the overall model [64]. The fusion could also occur at the decision level. For example, each modality trains one model and the final decision is determined by combining the decisions from all models, such as a majority vote scheme [66].

The application of CV in prosthetic control takes a different angle: shared control. In a pioneer study [67], Markovic *et al.* mounted stereo cameras on the prosthesis. The prosthesis user only needs to elicit a simple on/off command using EMG, without the need to control different grip patterns. CV algorithms were used to perform the latter task. The scene in the field of view of the cameras was analysed, the properties of the target object (e.g. size, position and orientation) within the scene were estimated and grip pattern parameters (e.g. orientation, aperture and strength) were estimated accordingly.

### Outlook

Multimodal sensing has been shown to effectively improve overall system performance, including accuracy, efficiency and robustness. As each sensor modality has its advantages and limitations, the design of the specific fusion strategy is important and key to complementing each other and leveraging their power. Sensor fusion strategies should consider the issues not only in signal processing algorithms (software end), but also in sensor integration, synchronization and power management (hardware end). The full stack implementation of a multimodality sensing system from hardware to software and its real-time performance test would be necessary for future studies and deserves more attention.

## MUAPT: THE ULTIMATE MYOELECTRIC CONTROL?

For the first time in the 2014 review, direct neural control information embedded in the MUAPt was proposed as the ‘ultimate feature’ for myoelectric control [68]. The signal processing methods of extracting concurrently active MUAPt from surface EMG have been referred to as sEMG decomposition. It was not a novel concept prior to the 2010s, as it has been an important research tool for muscle neurophysiology and movement control. However, it was only in the past decade that MUAPt has been explicitly applied in myoelectric control and it is proposed as a highly robust HMI to achieve dexterous and intuitive control.

**Figure 3.**
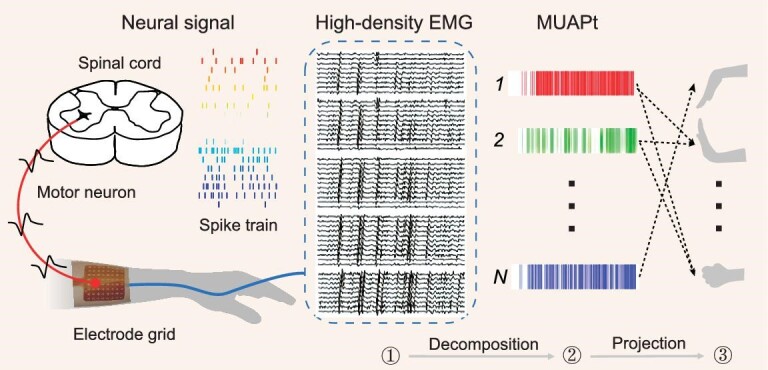
Illustration of MUAPt-based myoelectric control, adapted from Ref. [86] with permission.

A MUAPt-based myoelectric interface must consider two essential steps: decomposition and projection (Fig. 3). The former significantly affects the reliability and accuracy of identified MUAPts. The latter aims to establish a MUAPt-based control scheme by projecting or mapping the acquired MUAPts to the respective movement kinematics/kinetics, consequently translating them into control commands for devices.

### Decomposition of sEMG

Surface EMG decomposition methods are based on either blind source separation (BSS) or template matching [69,70]. In 2004, Holobar *et al*. proposed a convolution kernel compensation (CKC) algorithm to decompose HD-sEMG signals (>60 channels) into multiple MUAPts [71]. From then on, the CKC algorithm has been improved progressively by combining the natural gradient descent [72], K-means clustering [73] and fast independent component analysis (fICA) [74]. Currently, decomposition algorithms could identify MUAPts in full contraction levels and have been experimentally validated in several muscles and contraction conditions [75,76]. It should be noted that surface EMG decomposition identifies from ∼5 to ∼60 MUAPts per contraction. This number is relatively small, especially for high contraction levels with several hundreds of concurrently active MUs. The biggest limitation of the current decomposition methods is that they can only be applied in an offline fashion due to the high computational load. As such, achieving a real-time decomposition system for actual implementations in prosthetic control is of utmost importance. To this end, several decomposition methods have been generalized to online conditions through techniques such as pre-training the separation matrix [77]. At the same time, the adaptation strategies have been investigated to extend the decomposition algorithm in dynamic contractions [78,79]. Nevertheless, the real-time decomposition methods require further investigation in practical applications.

### Mapping from MUAPt to control parameters

The rapid development of sEMG decomposition algorithms has made its way into applications in HMIs in the past few years [68]. In order to translate the firing sequences of the motor unit action potentials (MUAPs) into neural information, the most common approach is simply counting the spikes in a sliding window, which reflects the instantaneous activation intensity of each motor unit [80,81]. Extracting the activation intensity of each motor unit individually during the projection may introduce undesired output fluctuation due to oscillations of single motor neurons. On the contrary, pooling the firings of all motor neurons together linearizes the relation between their synaptic input and the neural drive to the muscle, resulting in a more robust performance in the kinematics/kinetics projection [82]. Therefore, the discharge rate of the cumulative spike train (CST), which is calculated by combining all the identified MUAPts, is usually extracted to estimate the common efferent drive applied to the muscle. Generally, there is a strong linear correlation between the estimated neural drive and the movement kinematics/kinetics. It is noteworthy that the information loss may exist during the decomposition process since only a small portion of the motor unit pool could be identified. Including the neural features and the residual EMG signals would contribute to superior performance [83]. In this study, the spatial distribution of motor units from different muscles was leveraged, resulting in higher resolution in the multichannel MUAPs. The MUAP morphology could also be leveraged in classifying human movements, where a classification of >95% has been obtained for 12 hand gestures [84].

A more robust prediction model to project the neural features to human movements is also a key research direction. The stochastic nature of the order and number of identified MUAPts makes tracking motor units across motor tasks highly difficult, if not impossible. Therefore, several machine learning algorithms, such as support machine learning [84,85] and artificial neural networks [80], have been used to classify neural features into discrete gestures. In addition, dedicated calibration protocols were designed to correlate motor unit populations with specific motions [81,86]. However, the calibration method could only involve a few motions and is mainly used in continuous control of one or two DoFs. As to the continuous control, the neural features could be directly mapped to joint kinematics/kinetics with LR methods since the neural feature directly reflects the neural excitation [83]. Apart from the regression-based methods, the decomposition results have also been used to drive the musculoskeletal model [87]. The combination of MUAPt with the musculoskeletal model shows promising results in proportional control and provides the causal association between spinal motor neuron activity and joint moment control.

With decomposition and mapping ability improved, research efforts were made to realize online decoding. In 2017, Farina *et al*. applied these decoding techniques to myoelectric control on able-bodied subjects and upper-limb amputees using high-density EMG [68]. They mapped the MUAPts into control commands of multiple DoFs (two wrist DoFs and one elbow DoF) and the results presented superior performance compared with conventional EMG-based control methods. The real-time MUAPt-based control was first reported in 2018. Initially, the MUAPts were decoded in real time and used in the PRC approach, in which four hand gestures were classified with 91.9% accuracy [85]. In the SPC approach, the feasibility of online decomposition and projection has been validated in finger and wrist movements [81,88]. In pseudo-online analysis, the time delay of these applications was estimated to be <300 ms. The MUAPt-based control has also been validated in real-time interaction experiments. In 2021, Chen *et al.* demonstrated the superiority of real-time MUAPt-based SPC in a virtual target achievement control task, in which the subjects could control the two-DoF cursor simultaneously and proportionally, with higher accuracy and efficiency compared with conventional control schemes [86]. In 2022, Guerra *et al.* demonstrated that the MUAPt-based control led to high reproducibility and separability between finger contractions, as evidenced by the high task completion rate (>93% for 10 tasks) in a relatively short time (∼1.81 s per task) [80].

### Outlook

MUAPt-based myoelectric control has shown promising results in human movement decoding. However, there are still several limitations. The main limitation lies in the decomposition techniques. The limited number and uncertainty of identified MUAPts prohibit the projection of neural features to complex tasks such as the continuous movements of three or more DoFs. Also, the current decomposition techniques may fail to decode neural activities in dynamic movements and long-term applications because most decomposition algorithms assume a stationary mixing process. The current decomposition algorithms cannot accommodate changing motor unit recruitment after the initialization phase. The online decomposition methods with adaptation capacity to non-stationary conditions should be another research direction. Another limitation is the wearable integration of the decomposition system. Compared with the conventional EMG-based method, either in PRC or SPC, the hardware requirement and experimental set-up for MUAPt-based control are usually complex and cumbersome. More investigation is required to reduce the system complexity and streamline the experimental protocol.

## THE NEW HAMMER: DL?

One of the biggest ‘misses’ in the two reviews a decade ago was the explosion of DL in the 2010s. Compared with the traditional algorithms discussed so far, DL methods have unique feature-free or end-to-end learning characteristics. Feature extraction was performed implicitly through deep network structures. DL methods have increasingly become an important tool in all aspects of sEMG and myoelectric control research, demonstrating superior performance to traditional algorithms with hand-crafted features [89].

### DL algorithms for PRC

To address the challenge of parallelizing learning algorithms for deep architectures, Deng *et al*. proposed the deep stacking network (DSN) in 2012 [90]. The experimental results indicated that the DSN algorithm performed better than traditional deep neural networks. Moreover, in 2013, Hutchinson *et al*. proposed the tensor deep stacking network (T-DSN) to further improve training efficiency [91]. In 2015, Shim *et al*. proposed the deep belief network (DBN) algorithm [92]. Compared with back propagation algorithm, the DBN algorithm solved the problem of overfitting in the training model. The results showed that the classification accuracy of the DBN was higher than that of linear discriminant analysis (LDA) and SVM. Geng *et al*. used a convolutional neural network (CNN) to process sEMG for gesture recognition [93]. Their result demonstrated that CNN attained higher classification accuracy than LDA, SVM and other classification methods. Hu *et al*. proposed a hybrid model, combining a CNN and recurrent neural network (RNN) for EMG PRC [94]. The model achieved superior classification performance with more discrete electrode EMG and HD-sEMG data sets than traditional models. Despite its high computational requirement, DL methods were also extended to online protocols. Simão *et al*. compared the online PRC performance of an RNN, gated recurrent unit (GRU), feed-forward neural network and long short-term memory (LSTM) network on public EMG data sets [95]. The results showed that although the classification accuracy was similar, the training efficiency of LSTM and GRU was higher. Also using LSTM, Sun *et al*. demonstrated the feasibility of using the heterogeneous dilation of LSTM for transient-phase motion identification from high-density surface electromyography (HD-sEMG) [96].

### DL algorithms for SPC

DL methods have been investigated for SPC as well. Xia *et al*. proposed a combined CNN and RNN model to predict the upper-limb movements from sEMG signals [97]. The results showed that the prediction accuracy of the model was higher than that of the support vector regression (SVR) and CNN. A regression CNN model was applied to EMG in order to realize the SPC of two-DoF wrist movements [98]. The results showed that the regression CNN model could accurately extract the control information with superior performance. Yang *et al*. further proposed a CNN-based decoding approach that could realize SPC of three-DoF wrist movements [99,100]. The study compared the proposed approach with traditional regression methods, such as SVR. The results demonstrated that the CNN-based method significantly outperformed SVR.

Moving further distal, Guo *et al*. proposed an LSTM-based network to simultaneously estimate finger angles from sEMG signals [101]. The proposed approach not only achieved good performance but also showed great potential for real-time operation. Also working on finger SPC, an attentional network was proposed by Geng *et al.* and the results indicated that the proposed model significantly enhanced the finger movement estimation performance with respect to the LSTM and the Sparse Pseudo-input Gaussian processes [102].

### DL for better robustness

The feature-free characteristics of DL methods are ideal for robustness and adaptive processing. Ameri *et al*. proposed a supervised adaptation method based on CNN transfer learning (CNN-TL) to improve the EMG PRC robustness of electrode shift [50]. The experimental results showed that the proposed network enhanced the EMG PRC robustness against electrode shift. In a similar study on robustness, Shi *et al*. proposed a CNN-based multi-task dual-stream supervised domain adaptation (MDSDA) network to improve EMG PRC robustness and adaptability [103]. The results demonstrated that MDSDA significantly improved the robustness and adaptability of EMG PRC when compared with CNN and fine-tuning. For deep transfer learning, CNN [104] and RNN [105] provided a new strategy for improving the robustness of the myoelectric control: from explicit feature engineering to implicit (and automatic) feature learning. There were mainly two types of deep transfer learning structures. One updated a portion of the network with other parts frozen and the other calculated the domain-invariant feature via domain alignment.

### DL for decomposition and MUAPt mapping

Researchers in sEMG decomposition and MUAPt mapping have also investigated DL algorithms in the last few years, demonstrating exciting new opportunities.

In 2021, Clarke *et al*. used a GRU network to decompose both simulated and experimental high-density EMG signals [106]. The GRU network was trained with the output of the gradient CKC (gCKC). The GRU network outperformed gCKC at low signal-to-noise ratios, proving superior performance in generalizing to unseen data. Wen *et al*. proposed a novel approach to estimate the neural drive using a CNN to extract the CST [107]. The CNN estimated the CST with a correlation coefficient of >0.96 and normalized the root mean square error to <7% with respect to the BSS (golden standard) in each scenario. From the MUAPt mapping aspect, Yu *et al*. proposed an MU-specific image-based scheme for wrist torque estimation with a CNN. The proposed method outperformed three conventional and DL regression approaches [108].

### Outlook 

Although the DL-based EMG interface has become a research hot spot in the past decade, there are still several challenges in this direction, such as the high computational cost, the substantial data dependency, and the lack of interpretability. Firstly, even though powerful computing resource is available in the laboratory setting, it is not available in portable devices with a reasonable cost, not to mention the power consumption. This limits the practical applications of the DL-based EMG interface. Developing an embedded system with a low-power consumption GPU is a potential solution in the future. Secondly, a massive amount of data is needed to optimize the parameters of the DL method to ensure its good performance. In future works, data augmentation and transfer learning methods can be employed to increase the amount of data and improve stability with fewer data sampling points. Last but not least, the internal layer of most DL methods has neither physical nor physiological meaning that can be used to interpret the underlying physiological processes. Improving the interpretability of the DL methods would be an important future research direction to better implement the DL methods into the field of EMG interface.

## BETTER ACCESSIBILITY AND INCLUSIVITY: OPEN-ACCESS EMG DATABASES

Prior to the 2010s, different research groups in the myoelectric control area would report sEMG algorithms, with results obtained from data sets from their respective labs. These data sets were usually not available to researchers outside the respective lab. It was difficult to objectively compare the performances of different algorithms and created an obstacle for researchers who do not have access to EMG instrumentation or lack necessary human-participant research experiences. To foster a more inclusive research community and facilitate better cross-pollination from other researchers, making surface EMG data sets openly available has become increasingly popular in the myoelectric control research community. The Ninapro [109], a meta-database with sEMG, joint kinematics and more from both able-bodied subjects and amputees, is probably the most popular sEMG database currently in the literature. Many researchers, who would otherwise not be able to conduct sEMG-related research, have published their innovative research for both PRC and SPC using data sets from Ninapro. The data sets also facilitated objective comparison of different algorithms. More diverse data sets, some with unique features, e.g. large subject pools, multisessions and HD-EMG, are being published constantly. At the same time, the quality and consistency of the open-access data sets will be constantly improved.

## NON-INVASIVE BCI IN NEURAL PROSTHETICS AND BIO-ROBOTICS CONTROL

In the past decade, sEMG has demonstrated its power in prosthetic control, but still faces great challenges for trans-humeral amputees. Differently from sEMG signals from the central nervous system contain the source information of human intentions. BCI technology decodes human intentions from signals, including electroencephalography (EEG), magnetoencephalography (MEG) and functional magnetic resonance imaging (fMRI). People with muscular diseases such as amyotrophic lateral sclerosis might have normal brain signals but cannot generate effective EMG signals. Thus BCI bypasses the peripheral nervous system and builds a direct interface from the brain to the external devices [110]. But we restrict our review scope to EEG because EEG seems to be the most viable modality fusing with sEMG to solve the great challenges of restoring motion function for tran-humeral amputees [111].

Brain-controlled robotics based on BCI technology has seen a flowering of research in the past decade and is applied in assisting and rehabilitating human subjects. Successful applications include, but are not limited to, BCI-controlled neuroprosthetics, robotic arms [112] and quadcopters [113]. However, these demonstrations are still limited in well-controlled lab environments [110]. BCI technology is still in its infancy because the acquired signals are usually so weak that they are easily contaminated by for example sEMG signals. With the rapid development of new sensors and decoding methods, BCI has become a main branch of neuro-interfaces and has attracted more attention from academia and industry. Naturally, BCI is an essential part of bio-robotics research. The fuse of sEMG and EEG is emerging as research interests to provide neural prosthetic control for elbow-above amputees [114,115].

**Figure 4.**
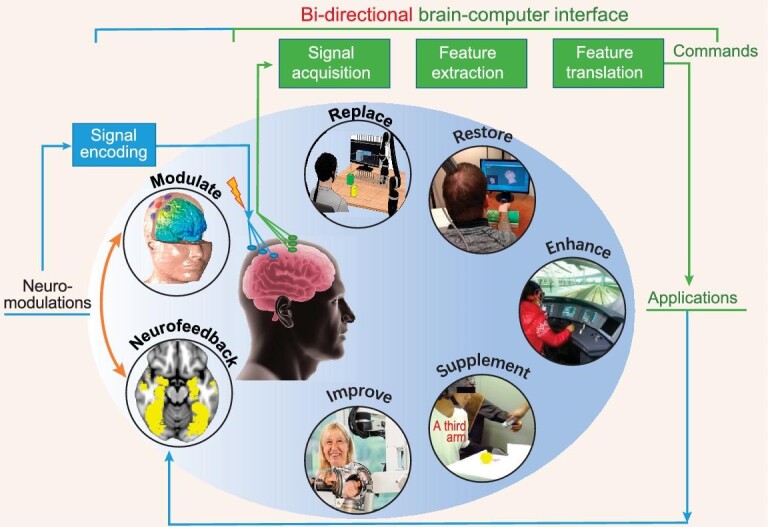
Schematics of brain–computer interface (BCI) systems, adapted from Ref. [110].

For a mind-to-device BCI, brain signals produced in response to various events are recorded from the scalp, from the cortical surface or from within the brain. These signals are analysed to extract features correlating with the user's intent. These features are then translated into commands that control application devices that replace, restore, enhance, supplement or improve natural central nervous system (CNS) outputs, as shown in Fig. 4.

### Signal sources

Various technologies have been developed to measure brain activities, including EEG, MEG, fMRI and functional near-infrared spectroscopy (fNIRS). A BCI could be categorized according to its signal sources, such as EEG and MEG. EEG reflects the neuronal and electrophysiological signals, and is measured by using specific instrumentation. EEG is widely used since it is relatively easy to acquire compared with MEG and fMRI, and is directly related to the neuronal activities of the human brain. Furthermore, EEG has a high temporal resolution compared with fMRI and fNIRS. In a broad sense, EEG could be acquired invasively and noninvasively. EEG usually refers to the electrical activities measured from the scalp, i.e. scalp EEG. Other invasive methods are not within the scope of this review. An excellent review of these methods can be found in [116]. Scalp EEG usually detects the average electrical activities of tens of millions of neurons beneath the surface electrodes. The electric activities of these neurons have to pass through various soft brain tissues and the skull before they can be detected by EEG electrodes placed on the scalp surface. As a result, EEG signals have a relatively low spatial resolution [117]. Recent attempts to increase the spatial resolution of EEG using source imaging have facilitated further development of EEG-based BCI. It has been demonstrated that single hand direction and wrist movement could be decoded in source space with relatively high accuracy after using the source imaging approaches [118].

### BCI modalities

#### Exogenous BCIs

A BCI could be built based on either exogenous stimulus or endogenous events. Since both exogenous BCIs [115] and endogenous BCI [114] how demonstrations of prosthetic control in combination with sEMG, both exogenous BCIs and endogenous BCI are reviewed, respectively. Visual stimulus is the most-applied exogenous stimulus. P300 and steady-state visual evoked potential (SSVEP) are commonly studied experimental paradigms based on visual stimulus. P300 occurs in the ‘oddball paradigm’, i.e. a large positive electrical response occurs at ∼300 ms after a rare stimulus appears in the subject's field of view. Researchers have designed P300-based robotic control applications [119]. Since P300 has a significant characteristic in the time domain, spatial filters such as xDAWN were proposed and have proven useful for improving the classification of P300. An improved version, axDAWN, was proposed as a better alternative since it adaptively updates the spatial filter parameters [120].

SSVEP is another type of visual stimuli response. When an individual pays attention to a visual stimulus with different repetitive frequencies (usually between 4 and 60 Hz), a large rhythmic EEG will be generated, namely SSVEP. The frequency of SSVEP usually contains both the stimulus frequency and their harmonics, and its signal-to-noise ratio (SNR) is usually sufficiently high such that it could be detected within a few hundred milliseconds after the stimulus onset. Thus, the SSVEP paradigm has been successfully applied for robotic device control [121]. For the SSVEP paradigm, canonical correlation analysis and its variants, such as filter bank canonical correlation analysis [122], are widely adopted in various SSVEP-based applications. These methods find projections such that the correlation between the projected raw signals and the template sinusoid signals is maximized. One of the highly attractive features of these methods is that they are essentially unsupervised and can be deployed without training data. More recently, a task-related component analysis (TRCA) approach was proposed and showed superiority in enhancing the detection accuracy of SSVEP [123]. However, the trade-off is that TRCA is a supervised approach and needs calibration data to set up the template signals.

#### Endogenous BCIs

Differently from the above exogenous BCIs, motor imagery (MI) or motor execution (ME) as a paradigm of the endogenous event is a mental rehearsal or execution of the actual movement [110]. MI tasks generate abundant movement-related cortical activations that do not require overt movement. It is especially useful for people who do not possess the normal pathway to control their muscles anymore. For example, people who suffer from stroke could use MI for functional rehabilitation [124]; amputees or people with spinal cord injuries could use MI to control prostheses [125]. However, it is still a highly challenging task to effectively extract useful information from the EEG to control the high DoF of the robotic device. Currently, most MI-related studies still focus on binary classification problems such as discriminating the left-hand versus right-hand imagination. New experimental paradigms such as investigating the continuous trajectory tracking have demonstrated the possibility of two-DoF real-time decoding of human motor intention [126]. Although it is still far from the objective of building a robust interface for dexterous neural prostheses control, this type of work sheds light on the future development of neural interfaces based on decoding human motor imagination.

The sensorimotor area is the most relevant area for MI and ME activities of limbs and the tongue. Thus the EEG from this area is highly correlated with the decoding performance of MI- and ME-based BCI. However, electrodes from other brain areas might contain useful information as well. The common spatial pattern approach and its improved versions (such as the filter bank common spatial pattern [127]) were proved to be effective spatial filtering methods for obtaining the optimized weights of a large number of electrodes and solving a binary classification problem [128]. After the spatial filtering, further feature extractions would be performed. To this end, two types of EEG signal modalities have been explored. Sensorimotor rhythm (SMR), a rhythmic change in alpha and beta band power in EEG over the sensorimotor area, has been extensively explored [128]. For example, Meng *et al*. demonstrated a non-invasive SMR-based BCI system for controlling a robotic arm; people in this experiment operated a robotic arm to reach and grasp objects to finish complex tasks in a 3D environment [112]. An alternative signal modality to SMR for MI/ME BCIs is movement-related cortical potential (MRCP), a slow cortical potential from the motor area. A randomized control clinical trial showed that an MRCP-based BCI induced fast-evolving and task-specific cortical plasticity in subacute stroke patients with a better clinical outcome than the current best clinical practice [129]. Laplacian filtering and locality preserving projection (LPP), a manifold dimensional reduction method, were instrumental for MRCP-based BCI [130].

In the past decade, Riemannian approaches were introduced into the classification of MI-based BCI in the recent decade. It blurs the boundary between feature extraction and classification [131]. The approach discriminates the covariance matrix of the EEG signals in a Riemannian manifold where the tangent space is equipped with an inner product. This method has been applied in both SMR [132] and MRCP modalities [133]. Thus, complete computation rules are set in this manifold. However, computation load is still an issue for such kinds of new decoding algorithms. Additionally, there is no physiological interpretation for such kinds of decoding.

#### Applications and future perspectives

In the past, BCI technology has experienced a decade of fast development in every aspect from signal acquisition, experimental paradigms to decoding approaches. High-density dry EEG electrodes with wireless communication are emerging and accelerating the development of portable BCI applications [121]. SSVEP-, MI-based BCI or the combination of both modalities has shown exciting prototypes of mind control of robotic devices, including neural prostheses, robotic arms, wheelchairs and quadcopters [110]. Novel hybrid modality BCI induced both SSVEP in the occipital area and SMR in the sensorimotor area, providing great potential for rehabilitation applications [134].

These latest developments have generally translated a human's intentions into manageable commands that robots could operate. Such translation of human intentions is meaningful to those who have lost normal communication channels and mobility. However, it is important to note that these translations differ from the EMG-driven prosthetic control discussed above and the invasive BCI control of neural prostheses, which usually decode the movement-related kinematic parameters directly [125]. However, these BCI modalities might provide a more robust candicate to supplement the sEMG-based myoelectric control for elbow-above amputees.

Although BCI as a neural interface has demonstrated its exciting prospects in robotic device control, many issues still must be sufficiently addressed before this technology can operate outside laboratory conditions. For example, BCI is a closed-loop system in which both the subject and the decoding approaches could adaptively change their status and parameters. Attaining a balance between the user and decoding algorithm adaptation [135] is necessary. Furthermore, the number of output DoFs and the decoding efficiency of current BCIs are still far behind in the need for dexterous manipulation of neural prostheses. On the one hand, more effort has to be made to improve the performance of the neural interface; on the other hand, a shared control strategy that fuses the advantage of BCI control and the intelligence of machines might be an alternative solution to solving such kinds of complex manipulation problems [136].

## DISCUSSION AND SUMMARY

Since the 2012 review, the dichotomy between academia and industry state-of-the-art of myoelectric control has been significantly bridged by a momentous shift in research focus and cross-sector collaboration. Prostheses with PRC algorithms embedded, such as Coapt [7] and Myo Plus Pattern Recognition [8], have become commercially available. While users and other stakeholders are evaluating their clinical and commercial viability, significant research efforts are being invested worldwide with the ultimate yet elusive goal of making upper-limb prostheses as natural as the biological arm.

The four challenges identified a decade ago are still relevant on two levels. First, none of the four challenges can be considered ‘solved’ and they deserve more research. More importantly, most studies have exclusively addressed one of the challenges. However, these issues are not isolated, but are closely coupled aspects of the overall system. For example, algorithms in both PRC and SPC schemes face robustness challenges; the choice of EMG control schemes (tDC, PRC or SPC) should be considered in developing multimodality sensing approaches; the design process of the sensory feedback method should be mindful of the robustness issue as early as possible. Therefore, more holistic approaches must be taken in future studies.

sEMG decomposition and MUAPt-based control provide a novel and exciting new avenue to reaching the ultimate goal. As discussed above, decomposing in dynamic contractions, real-time capability and robustness should be focused on in future research. Another powerful tool made available for myoelectric control researchers in the past decade was DL algorithms. As outlined above, DL algorithms have demonstrated their prowess in almost all aspects of myoelectric research, from PRC, SPC, to robustness and decomposition. Of course, DL comes with its limitations, the most notable of which is its insatiable appetite for the amount of data. To this end, the increasing amount of open-access myoelectric control data sets has provided invaluable ‘fuel’ for researchers using DL algorithms (and traditional methods) while making the global research community increasingly inclusive. We also discussed the relevant research on another neural interfacing technology: non-invasive BCI. Most research in myoelectric control and BCI have been conducted in a silo, i.e. EMG and EEG were used almost separately in current research. The clearly complementary neural information embedded in these two signal modalities calls for a synergistic approach to fully leverage their respective advantages in a tiered and application-specific approach.

While the current review is limited to the surface and noninvasive methods, invasive methods to acquire EMG can also be used as an alternative interface to sEMG. Invasive methods, either intra-muscular or epimysial, provide significantly higher signal bandwidth and better physical separation between channels than sEMG because the electrodes are much closer to the signal source and do not suffer from the volume conductor effect of the skin. As such, invasive methods allow for different control paradigm, such as direct control of multiple DOFs. However, invasive methods still pose much higher risks and therefore difficult to scale up for wider spread application as non-invasive methods.

There is still a long march before the state-of-the-art in academic research can be translated into commercially and clinically viable prosthetic products that provide meaningful and affordable functional replacements for amputees. The focus of the current review is on the innovation `soft' side of the prosthetic technology. On the ‘hard’ side, i.e. mechanical structure, mechatronics and electronics design, has also seen significant stride in the past decades, such as superb structure design for high anthropomorphic features[30] and integration of flexible electrodes and electronics [136]. The synergistic combination of innovations from both `soft' and `hard' aspects will required for ultimate goal of human-like prostheses that are intuitively functional, clinically practical and financially viable.
